# Knowledge, beliefs, and perceptions of Turkish vitiligo patients regarding their condition*

**DOI:** 10.1590/abd1806-4841.20165060

**Published:** 2016

**Authors:** Ilteris Oguz Topal, Hatice Duman, Ozgur Emek Kocaturk Goncu, Mustafa Durmuscan, Sule Gungor, Pelin Kuteyla Ulkumen

**Affiliations:** 1 Okmeydani Training and Research Hospital – Istanbul, Turkey; 2 Adana Public Health Laboratory – Adana, Turkey

**Keywords:** Anxiety, Knowledge, Perception, Vitiligo

## Abstract

**BACKGROUND:**

Vitiligo is an acquired pigmentary skin disorder that affects 0.5% to 2.0% of the population.

**OBJECTIVE:**

Patients' knowledge, opinions, and attitudes about vitiligo were evaluated.

**METHODS:**

The team conducted a cross-sectional, descriptive, prospective study between June 2014 and May 2015. The study included 100 patients aged over 12 years who were diagnosed with vitiligo. A questionnaire including items on knowledge, opinions, and beliefs about vitiligo and the Illness Perception Questionnaire (IPQ) were filled out by the patients, and the results were analyzed.

**RESULTS:**

In total, 100 (58 female, 42 male) patients were included in the study. Of them, 74% knew the name of their disease, 90% thought that vitiligo was not contagious, 48% reported that they obtained information on the disease from a doctor, and 69% believed they had adequate information on vitiligo. Eighty percent reported no negative effects from vitiligo on relationships with friends or family. It was believed that stress, excessive sun exposure, and heredity were causes of vitiligo, according to 84%, 37%, and 22% of the patients, respectively. Thirty-six patients (36%) believed that their illness was a serious disease and 35% deemed that it did not have a major impact on their lives.

**CONCLUSIONS:**

Our results show that vitiligo patients were generally highly aware of their condition. The disease did not negatively affect patient opinions or attitudes about vitiligo. The authors believe that improving patient-physician communication will impact positively on the course of the disease.

## INTRODUCTION

Vitiligo is a common depigmenting skin disorder characterized by acquired, idiopathic, progressive, circumscribed hypomelanosis of the skin and hair. It occurs worldwide, with an incidence of 0.5% to 2.0%.^1^

Skin diseases are often visible to others, and many skin disorders may entail significant psychosocial consequences. Vitiligo is a social stigma and is associated with a decreased quality of life, especially when lesions are located over the face; thus, it may affect the quality of social and personal life depending on the patient's perception.^2^

Some studies on the anxiety and depression associated with vitiligo were performed in Turkey.^3^ In one of them, the rate of psychiatric morbidity was higher in vitiligo patients than in healthy control subjects.^4^ However, no reported study has evaluated the beliefs, knowledge, and opinions about vitiligo among affected patients. This study aimed to evaluate affected patients' knowledge, attitudes, perceptions, and opinions about vitiligo.

## METHODS

### Participants, questionnaire and data collection

A cross-sectional, descriptive, prospective study was conducted at the dermatology outpatient clinic of the Okmeydani Training and Research Hospital from June 2014 to May 2015. In total, 100 consecutive patients who had a diagnosis of vitiligo confirmed by a dermatologist were included in the study. A questionnaire including items on knowledge, opinions and attitudes about vitiligo was filled out by the patients, and the results were analyzed statistically. Patients were subdivided into the following three subgroups, according to age: the young adult group <30 years of age (n = 41),the adult group aged between 30 and 49 years (n = 39) and the elderly group >50 years (n = 20).

The study was approved by the medical ethics committee of the Okmeydani Training and Research Hospital. All participants gave written informed consent to participate in the study.

### Questionnaire

The Illness Perception Questionnaire (IPQ) comprises 19 questions that require approximately 4 minutes to be answered.^5^ It consists of two parts. The first concerns personal information, such as age, sex, level of education, and marital status, while the second involves perceptions and opinions about vitiligo. The IPQ was completed by patients aged over 12.

The IPQ has been used in patients with cardiac disease, psoriasis, and chronic fatigue syndrome.^6-8^ Because vitiligo is an asymptomatic disease, the team did not use the subscale of "symptoms" in the study. The second part of the questionnaire comprises four subscales: the cause subscale (17 items) measures personal ideas about the causes of vitiligo, the timeline subscale (3 items) deals with perceptions regardingdisease duration, the consequences subscale (6 items) treats the expected effects and outcomes of the illness, and the cure/control subscale (6 items) details beliefs about recovery from or control of the condition. Furthermore, beliefs about the possibility of the disease being contagious, its emotional effects, information on vitiligo, major sources of information on the disease, and effects on school performance, were analyzed in patients. The reliability and validity of the questionnaire scale for Turkish patients was reported by Kocaman *et al.*^9^

### Data analysis and statistics

SPSS software (ver. 17; SPSS Inc., Chicago, IL) was used for statistical analyses. The descriptive analysis was presented in tables as mean (SD) and median (minimum-maximum) for numeric data and frequency (n) and percentage (%) for categorical data.

The Chi-Square test and Fisher's exact test were used to assess associations between categorical variables. The Mann-Whitney U test was used to compare numerical variables. Statistical significance was set at p < 0.05.

## RESULTS

### Sociodemographic data

In total, 100 vitiligo patients (42 male, 58 female) were recruited. The patients' ages ranged from 13 to 78 (35.4 ± 14.3) years. The age at onset ranged from 4 to 73 (28.9 ± 14.4) years (Table 1). Most patients had acrofacial vitiligo (37%), followed by focal vitiligo (32%), generalized vitiligo (28%) and universal vitiligo (3%). Of the patients, 63 (63%) were married, while 37 (37%) were unmarried; 85 (85%) patients had a high school education or lower level, while 15 (15%) patients had a college education or higher (Table 1).

**Table 1:** Demographic characteristics of the patients based on sex and education level

Variable	Total (n=100)	Female (n=58)	Male (n=42)	Patients with lower educational level (n=85)	Patients with higher educational level (n=15)
	Mean ± SD/ Median (Min-Max)				
Age (year)	35.4±14.3/	34.7±14/	36.3±14.9/	36.7±1/	28±6.6/
	32(13-78)	32.5(13-67)	31(15-78)	35(13-78)	29(20-43)
Age of onset of disease (year)	28.9±14.4/	28.9±14.6/	28.9±14.2/	30±15/	22.7±8.2 /
	29(4-73)	29(4-61)	28.5(5-73)	0(4-73)	23 (6-39)
Diseaseduration (months)	80.7±84.7/	74.8±85.4/	88.9±84/	84±87.1/	62±68.9 /
	48(1-360)	39(1-360)	60(2-360)	60(1-360)	60(5-240)

SD: Standard deviation, min: minimum, max: maximum

### Beliefs about the cause of the disease

Table 2 shows the percentage of patients "agreeing" with each cause item. The most common belief regarding the cause of the disease was stress (84%), followed by excessive sun exposure (37%) and heredity (22%). Patients who had a higher level of education believed more commonly than those with lower educational levels (p = 0.014) that vitiligo developed because of altered immunity. Male patients were more likely to think that vitiligo developed because of excessive work than female patients (p = 0.010).

**Table 2:** Beliefs of vitiligo patients about the causes of their illness*and analysis according to sex and educational level

Cause	Total (n=100)	Female (n=58)		Male (n=42)		p	Patients with lower education level (n=85)		Patients with higher education level (n=15)		p
		n	%	n	%		n	%	n	%	
**Stress**	84	49	84.5%	35	83.3%	1.000^a^	71	83.5%	13	86.7%	1.000^b^
**Excessivesunexposure**	37	21	36.2%	16	38.1%	1.000^a^	32	37.6%	5	33.3%	0.977^a^
**Heredity**	22	15	25.9%	7	16.7%	0.395^a^	17	20.0%	5	33.3%	0.310^b^
**Excessivework**	20	6	10.3%	14	33.3%	0.010^a^	17	20.0%	3	20.0%	1.000^b^
**Family problems**	17	9	15.5%	8	19.0%	0.846^a^	16	18.8%	1	6.7%	0.456^b^
**Altered immunity**	16	6	10.3%	10	23.8%	0.124^a^	10	11.8%	6	40.0%	0.014^b^
**Fate**	13	7	12.1%	6	14.3%	0.981^a^	13	15.3%	0	0.0%	0.207^b^
**Chance**	10	6	10.3%	4	9.5%	1.000^b^	9	10.6%	1	6.7%	1.000^b^
**Diet**	9	5	8.6%	4	9.5%	1.000^b^	8	9.4%	1	6.7%	1.000^b^
**Evileye**	7	4	6.9%	3	7.1%	1.000^b^	6	7.1%	1	6.7%	1.000^b^
**Cigarettes**	6	1	1.7%	5	11.9%	0.080^b^	6	7.1%	0	0.0%	0.587^b^
**Jinn**	5	4	6.9%	1	2.4%	0.395^b^	5	5.9%	0	0.0%	1.000^b^
**Virus**	3	2	3.4%	1	2.4%	1.000^b^	3	3.5%	0	0.0%	1.000^b^
**Poor medical care**	2	1	1.7%	1	2.4%	1.000^b^	2	2.4%	0	0.0%	1.000^b^
**Alcohol**	2	0	0.0%	2	4.8%	0.174^b^	2	2.4%	0	0.0%	1.000^b^
**Accident**	1	0	0.0%	1	2.4%	0.420^b^	1	1.2%	0	0.0%	1.000^b^
**Sorcery**	0	0	0.0%	0	0.0%		0	0.0%	0	0.0%	

a p values were calculated by Chi-Square test;

b p values were calculated by Fisher’s exact test;

* Multiple answers were possible for this question.

### Beliefs about consequences

Table 3 displays the percentage of patients "agreeing" with different consequence items. Thirty-six (36%) patients believed that their illness was a serious disease, and 35% believedthat their illness did not have a major impact on their lives. No association was found between the beliefs about consequences and educational level or age (p > 0.05). Female patients were more likely to think that vitiligo was a serious disease (p = 0.031). Male vitiligo patients thought that their illness had no major impact on their lives (p = 0.041).

**Table 3:** Patients’ beliefs about the consequences of having vitiligo and analysis according to sex and educational level

Belief	Total (n=100)	Female (n=58)	Male (n=42)	p	Patients with lower education level(n=85)	Patients with higher education level(n=15)	p
My vitiligo is a serious condition	36	44.8%	23.8%	0.031^a^	37.6%	26.7%	0.600^a^
My illness does not have much effect on my life	35	25.9%	47.6%	0.041^a^	32.9%	46.7%	0.463^a^
My vitiligo has had a major impact on my life	27	32.8%	19.0%	0.195^a^	27.1%	26.7%	1.000^b^
My vitiligo causes difficulties for those who are close to me	15	15.5%	14.3%	1.000^a^	16.5%	6.7%	0.457^b^
My vitiligo has become easier to live with	13	10.3%	16.7%	0.531^a^	11.8%	20.0%	0.407^b^
My vitiligo has serious economic and financial consequences	3	1.7%	4.8%	0.571^b^	3.5%	0.0%	1.000^b^

a p values were calculated by Chi-Square test;

b p values were calculated by Fisher's exact test.

### Beliefs about recurrence or chronicity

Among the patients, 44% believed their illness was likely to be a long-term condition. However, there was no association between this belief and the patients'educational level or sex. Sixty-six percent of the patients in the young adult group deemed that their illness was likely to be a long-term condition (p < 0.001). Twenty-four patients thought that their illness would last a short time. The patients who believed their illness was likely to be a long-term condition were more likely to experience a longer duration of disease (60 months (1-360 months), p = 0.015) (Table 4). Sixty percent of the patients in the elderly group thought that their illness would be permanent (p = 0.009).

**Table 4:** Effects of disease duration on patients' beliefs about recurrence or chronicity and patients’ beliefs about cure and control

Variable	Disease duration (months) Median (Min-Max)	p
Patients who believe their illness will last a short time (n=24, $)	21 (1-192)	0.015
Patients who believe their illness is likely to be permanent or last for a long time (n=76, v)	60 (1-360)	0.015
Patients who believe their vitiligo will somehow improve in time* (n=82, ‚)	36 (1-360)	0.006
Patients who believe very little can be done to improve their vitiligo (n=18, )	84 (12-360)	0.006

The p-values were calculated by Mann-Whitney U test;

* Their treatment will be effective in curing my vitiligo or; There is a lot that I can do to control my vitiligo or; Recovery from their vitiligo is largely dependent on fate or chance or; What they do can determine whether their vitiligo gets better or worse.

### Beliefs about cures and control

Table 5 presents the data on the patients' opinions about cures and control. Forty-seven percent believed their vitiligo would improve with time, while 18% believed very little could be done to improve their illness. However, these patients were more likely to experience a longer duration of disease (84 months (12-360 months), p = 0.006) (Table 4). Forty-five percent of the patients in the elderly group thought very little could be done to improve their illness (p = 0.002). Additionally, 30% of the elderly patients stated that what they did actions could determine whether their vitiligo got better or worse (p = 0.004). Forty-three percent of the patients in the adult group thought their treatment would be effective in curing their vitiligo (p = 0.032). No association was found between the beliefs about cures and control or clinical type of vitiligo. There was no association between beliefs about cures and control or patients' sex (p > 0.05).

**Table 5:** Patients' beliefs about cure and control and analysis according to sex and age

Belief	Total (n=100)	Female (n=58)	Male (n=42)	p	Young adultgroup (n=41)	Adultgroup (n=39)	Elderlygroup (n=20)	p
My vitiligo will improve in time	47	50.0%	42.9%	0.615^a^	48.8%	48.7%	40.0%	0.782^a^
My treatment will be effective in curing my vitiligo	32	34.5%	28.6%	0.683^a^	31.7%	43.6%	10.0%	0.032^a^
Very little can be done to improve my vitiligo	18	13.8%	23.8%	0.306^a^	14.6%	7.7%	45.0%	0.002^a^
Recovery from my vitiligo is largely dependent on fate or chance	16	13.8%	19.0%	0.666^a^	9.8%	23.1%	15.0%	0.265^a^
I can do a lot to control my vitiligo	14	12.1%	16.7%	0.717^a^	22.0%	12.8%	0.0%	0.065^a^
What I do can determine whether my vitiligo gets better or worse	10	6.9%	14.3%	0.314^b^	4.9%	5.1%	30.0%	0.004^a^

a p values were calculated by Chi-Square test;

b p values were calculated by Fisher’s exact test;

* Multiple answers were possible for this question.

### Understanding the disease

Seventy-four percentstated: "I know the name of my illness." Moreover, 90% thought that vitiligo was not contagious. There was no statistically significant difference between patients with lower educational levels and those with higher levels (p > 0.05). Sixty-nine percent reported having knowledge of their illness. Participants stated that their information sources for vitiligo were: a doctor (48%), the Internet (46%) and friends or family (7%). Furthermore, 91%indicated that the illness did not have a serious impact on their school performance, while 80% stated the disease did not impact on their social relationships, work, or school activities.

### Emotional effects

Forty-six percent of the patients reported that their illness made them feel anxious. However, there was no significant association between reports of anxiety and patients' sex or level of education. In addition, 28% reported that having the illness did not make them feel anxious. This finding was more prevalent in males than females (p = 0.032). Twenty-nine percent reported that the illness made them feel angry, while 12% indicated that they became depressed when thinking about their illness.

## DISCUSSION

Skin diseases affect a patient's appearance, social life, and emotional situation. These effects include psychological stress, embarrassment, and physical disability. Many studies have shown that psychiatric disorders occur frequently in patients with skin disorders.^5^

Vitiligo is characterized by hypopigmented skin lesions and can cause psychological stress, such as depression, anxiety, and problematic social relationships, which can lead to a significantly impaired quality of life.^10^ Psychiatric problems associated with vitiligo have been studied widely.^11^

However, the number of studies on patients' knowledge, attitudes, perceptions, and opinions about vitiligo is limited. It seems there are only two studies on this topic in the literature.^12,13^ The first, conducted by Firooz et al.^12^ in 2004, is a questionnaire comprising 25 questions about the causes, timeline, consequences, and control of the disease, administered to 80 vitiligo patients. They observed that 62.5% of the patients believed stress played a major role in their disease, while 31.3% thought their genetic background had an influence on their disease. A recent study by Al Ghamdi*et al.*^13^ analyzed Arabian vitiligo patients, where most patients (84%) believed in fate as a cause of vitiligo; stress, altered immunity, and heredity were reported as the other causes (33%, 26%, and 24% of the patients, respectively). A significant proportion of their patients (28%) believed that "evil eye" was responsible for their illness.^13^ Our patients deemed that the most common causes of the disease were stress (84%), excessive sun exposure (37%), heredity (22%) and excessive work (20%). Patients with higher educational levels believed more often than those with lower levels (p = 0.014) that altered immunity could cause vitiligo. The percentages of patients who thought that fate and evil eye could be causes of vitiligo were only 13% and 7%, respectively. These lower ratios may be due to cultural and regional differences. The authors think that despite the lower ratio of college education and higher among our patients, the ratios for Internet use and consulting a doctor were high, while the percentages of patients with beliefs about fate and evil eye as causes of vitiligo were therefore lower.

Similarly to the study by AlGhamdi *et al.*, beliefs about the contagiousness of vitiligo were striking; in our study, 90% of the patients thought that it was not contagious.^13^ There was no statistically significant difference between patients with lower versus higher educational levels (P > 0.05).

Firooz*et al.*^12^ reported that half of the vitiligo patients (49%) deemed their illness had a strong impact on their lives. Similarly, AlGhamdi *et al.*^13^ found this ratio to be 42%. Unlike these studies, our ratio was lower (27%).

Thirty-five percent of all patients said their illness did not have a major impact on their lives. Male vitiligo patients thought this more frequently than female patients (P = 0.041), who were more likely to think that vitiligo was a serious disease (P = 0.031). Studies carried out by Noh *et al.*^10^ showed that the quality of life of vitiligo patients was significantly impaired compared with that of healthy subjects. Females exhibited greater private body consciousness than males.^10^ This may be explained by women's greater awareness of cosmetic disfigurement. Thus, they may consider that vitiligo is an important disease. Our finding is consistent with previous psychological literature, which demonstrated a higher level of concern with physical appearance and the seriousness of the illness.

Regarding disease duration,the team found that 24 (24%) patients believed their illness would last a short time. In previous studies, only limited proportions of patients thought their illness would last a short period (10% in Saudi Arabia, 14% in Iran).^12,13^ Of our patients, 44% (versus 55% of Arabian patients) believed their illness was likely to be of a long, rather than temporary, duration. They also endured a longer duration of disease. With respect to the association between age and beliefs about recurrence or chronicity, our study found that elderly patients were more likely to think their illness would be permanent (p = 0.009). This belief may be related to their compliance with the disease.

Our patients were not hopeless about the cure and control of vitiligo, unlike in some previous studies. Indeed, 47% believed their illness would improve with time. In Saudi Arabia, 39% of vitiligo patients stated there was very little that could be done to improve their illness, which is similar to Iranian study results (41%).^12,13^ In our study, this ratio was only 18%. The authors believe that recent developments in medicine and new drugs in therapy may have affected patients' opinions.

Beliefs about the cure and control of vitiligo were independent of sex and clinical type of vitiligo. But compared with young patients, elderly patients were more likely to think very little could be done to improve their illness (P = 0.002). The authors think the low success rate of vitiligo treatment and low expectations regarding prognosis may be related to this belief.

A wide range of psychological characteristics have been reported in various cross-sectional surveys, including high depression/anxiety scores, obsessionality, social stigmatization and other psychosocial comorbidities experienced by vitiligo patients.^14^ Ahmed *et al.* identified major depression (35.7%), anxiety (23.5%), social phobia (19.0%) and sexual dysfunction (4.7%) in vitiligo patients.^15^The study by AlGhamdi *et al.* involved a higher proportion of depression^13^ (54%). Of our patients, 12% reported feeling depressed when thinking about their illness. In our patients, no significant association was uncovered between emotional effects and educational levels or sex. Twenty-eight percent of our patients reported that having this illness did not make them feel anxious. This finding was more prevalent in males than females (p = 0.032). Al Robaee noted that women were more embarrassed and self-conscious about their disease than men because it impaired their social life and personal relationships.^16^ Similar findings have been observed in several studies, concluding that disturbances in social lives and psychiatric morbidity are more frequent in female patients.^17^ Our findings are consistent with these reports.

In the UK, the results of a recent survey from a patient support group showed that most respondents obtained information about their disease from non-medical sources (83.0%), 12.5% from dermatologists, and 7.1% from their general practitioners.^16^ In our study, information on vitiligo was obtained most frequently from a doctor (48%) or the Internet (46%). Of our patients, 69% believed that information on vitiligo from these sources was adequate. Furthermore, more than half of our patients knew the name of their illness. Although we found that the proportion was lower for patients with higher educational levels, they had information about the disease and had visited a physician.

When the impact of the self-image of vitiligo was questioned, 80% of patients indicated that vitiligo did not have a serious impact on their relationships with family or friends. Moreover, vitiligo did not have a significant impact on work/school performance (91%).

## CONCLUSION

In conclusion, our results suggest that a majority of the patients had knowledge about their illness. They were also aware of the causes. In recent years, sources of information such as the Internet and medical publications have increased, so patients have more knowledge about their disease. It is known that vitiligo is not life-threatening. Thus, although some patients were anxious, they had positive ideas on cures and disease control. As physicians, attending to patients should improve their outlook on the prognosis and cure of the disease. Improving patient–physician communication will impact positively on the course of the disease.
